# Data Processing Pipeline for Lipid Profiling of Carotid Atherosclerotic Plaque with Mass Spectrometry Imaging

**DOI:** 10.1007/s13361-019-02254-y

**Published:** 2019-06-27

**Authors:** Mirjam Visscher, Astrid M. Moerman, Peter C. Burgers, Heleen M. M. Van Beusekom, Theo M. Luider, Hence J. M. Verhagen, Antonius F. W. Van der Steen, Kim Van der Heiden, Gijs Van Soest

**Affiliations:** 1 Department of Biomedical Engineering, Thorax Center, Erasmus MC, Rotterdam, The Netherlands; 2 Department of Neurology, Laboratory of Neuro-Oncology, Erasmus MC, Rotterdam, The Netherlands; 3 Department of Experimental Cardiology, Thorax Center, Erasmus MC, Rotterdam, The Netherlands; 4 Department of Vascular and Endovascular Surgery, Erasmus Medical Center, Rotterdam, The Netherlands; 5 Medical Delta, Delft, Rotterdam, The Netherlands; 6 Shenzhen Institutes of Advanced Technology, Chinese Academy of Sciences, Shenzhen, China

**Keywords:** Artherosclerosis, Plaque, Lipids, MALDI-MSI, Imaging, Method, Mass spectrometry, Vulnerable plaque, Endarterectomy, Carotid artery, Thrombus

## Abstract

Atherosclerosis is a lipid and inflammation-driven disease of the arteries that is characterized by gradual buildup of plaques in the vascular wall. A so-called vulnerable plaque, consisting of a lipid-rich necrotic core contained by a thin fibrous cap, may rupture and trigger thrombus formation, which can lead to ischemia in the heart (heart attack) or in the brain (stroke). In this study, we present a protocol to investigate the lipid composition of advanced human carotid plaques using matrix-assisted laser desorption ionization (MALDI) mass spectrometry imaging (MSI), providing a framework that should enable the discrimination of vulnerable from stable plaques based on lipid composition. We optimized the tissue preparation and imaging methods by systematically analyzing data from three specimens: two human carotid endarterectomy samples (advanced plaque) and one autopsy sample (early stage plaque). We show a robust data reduction method and evaluate the variability of the endarterectomy samples. We found diacylglycerols to be more abundant in a thrombotic area compared to other plaque areas and could distinguish advanced plaque from early stage plaque based on cholesteryl ester composition. We plan to use this systematic approach to analyze a larger dataset of carotid atherosclerotic plaques.

**Electronic supplementary material:**

The online version of this article (10.1007/s13361-019-02254-y) contains supplementary material, which is available to authorized users.

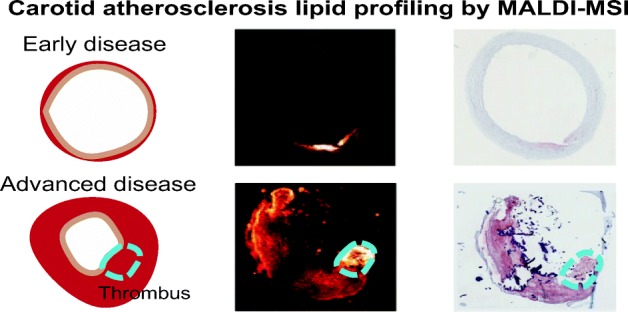

## Introduction

Atherosclerosis is the cause of more than 10 million deaths worldwide, and a major contributor to long-term morbidity and disability [1]. This disease of the artery walls is characterized by the gradual formation of depositions in the vascular wall that are called atherosclerotic plaques. Plaque growth is initiated by endothelial dysfunction and driven by impaired lipid transport mechanisms, which trigger an inflammatory response. Lipids thus play a key role in the pathogenesis of atherosclerosis. Besides a variety of lipid species, these plaques contain calcifications, fibrous tissue, smooth muscle cells, and necrotic material [2, 3]. Depending on the tissue composition and structure of the plaque, plaques can be either “stable” or ”vulnerable,” the latter means they are prone to rupture. Plaque rupture may trigger embolization of fragments of plaque tissue in the bloodstream, which, depending on the anatomical location of the plaque, can lead to stroke or myocardial infarction [3, 4]. The mechanisms driving the evolution of a stable plaque to a vulnerable phenotype are not known in detail, although it is clear that defective or unbalanced lipid transport plays a key role [5–9]. The recent LRP study (presented at TCT2018 [10]) demonstrated that coronary vessel segments with lipid-rich plaque have a > 4 times higher clinical event rate than vessel segments without such plaque, highlighting the importance of plaque lipid content for triggering ischemia. We aim to study whether the lipid profile differs between stable and vulnerable plaque types. To determine whether a plaque’s lipid profile can be correlated to plaque phenotype and, therefore, risk of rupture, variation between and within plaque samples needs to be assessed. In this study, we present a mass spectrometry imaging (MSI) method for systematically studying plaque lipid distribution at various stages of development. MSI is an emerging experimental technique in the molecular characterization of biological samples. Using MSI, the molecular composition of thin tissue sections can be imaged at a micron-scale resolution. As an initial step, MSI requires ionization of the tissue section. Different methods of ionization exist, including matrix-assisted laser desorption ionization (MALDI), a commonly used ionization method for biological samples [11]. The main advantages of MALDI are its ability to generate singly charged ions, which greatly simplifies analysis of mass spectra, and its ability of so-called soft ionization, preventing molecules from fragmenting during the ionization process [11]. MALDI-MSI is suitable for the detection of small molecules, such as lipids.

To date, few studies of (MALDI-)MSI on human arteries have been published [12–18]. They explored the parameter space of atherosclerotic plaque composition as sampled by MSI along various dimensions. Prior work examined different arterial beds, i.e., aorta [16], carotid [14, 15, 17], coronary arteries [13], femoral, or popliteal [12, 18], and differed in the ionization method used, i.e., MALDI [12, 16–18], secondary ion mass spectrometry (SIMS) [13, 14] or desorption electrospray ionization (DESI) [15]. Clinically relevant plaques, those that are likely to precipitate ischemic events, contain a variable mix of lipids [6]. In studying the role of various lipids [7, 19] in the atherosclerotic disease process, it is therefore important to optimize the analysis for accurately sampling the lipid spectrum, and to quantitatively understand the variance in measured ion abundances. We consequently chose to focus on lipid-rich plaques only, and moreover restricted ourselves to carotid plaques, as these are a major precursor of ischemic stroke [20].

Our aim is to develop a MALDI-MSI pipeline for the analysis of lipids in human carotid artery plaques, suitable for assessing the variation in lipid profile, both between different plaque specimens and within different locations in the same specimen. To that aim, we add the following insights to the existing MSI knowledge: (1) We describe how arterial tissue should be processed, such that the combination of MALDI-MSI and histological information enables contextualization of MSI data in terms of the known morphological features of plaque pathology. (2) We combine various MSI data analysis methods into a robust data processing pipeline that reliably reduces an MSI dataset into manageable lipid-specific results. We describe this pipeline in detail to encourage other researchers to follow similar pipelines in their own research. (3) We assess the reproducibility of measurements by calculating the coefficient of variation of MALDI-MSI measurement results of tissue sections that were located close together.

In addition, we discuss tentative observations in three human carotid plaques that illustrate the potential for imaging lipid biochemistry in atherosclerosis. This method is readily applicable to larger collections, limited by scan time, of carotid plaque specimens, allowing to assess the relation between the carotid plaque’s molecular lipid composition and plaque type using statistical methods.

## Materials and Experimental Methods

### Materials

DPBS (without calcium and magnesium), KP Haematoxyline mayer (VWRK4085–9002), Resorcin (1075930100), and SuperFrostPlus glass slides (76 × 26 mm) were purchased from VWR (The Netherlands). Eosine (HT110232-1 L), Oil Red O (00625-25G), Basic Fuchsin (857343-25G), Ferric(III) chloride (157740-100G), 2,5-dihydroxybenzoic acid (98%, 149,357-100G) and porcine type A gelatin were purchased from Sigma-Aldrich (The Netherlands). HPLC-grade acetone was purchased from Fisher Scientific (The Netherlands). Mouse anti-human CD31 (JC/70A, ab9498) and mouse anti-human CD68 (KP1, ab955) were purchased from Abcam (UK). EnVision + Kit (HRP, mouse, 1100 tests, 110 ml, K4001) was purchased from Agilent (the Netherlands). Lipid standards Cholest-5-en-3ß-yl heptadecanoate (CE 17:0) 1 mg/mL (700,186 M^−1^ mg) and 1,2-diheptadecanoyl-sn-glycero-3-phosphocholine (PC 17:0) 10 mg/mL (850360C-25 mg) were purchased from Avanti Polar Lipids (USA).

### Tissue Collection

Carotid endarterectomy (CEA) surgery was performed by carefully opening the outer wall of the carotid artery and excising the plaque by separating the intima and media. This protocol preserves an intact lumen and plaque morphology [21]. Two human carotid plaque specimens, harvested at carotid endarterectomy (CEA) surgery, were rinsed in phosphate-buffered saline (DPBS), snap frozen and stored at − 80 °C until further processing. A third carotid artery specimen, without apparent atherosclerotic disease, was collected at autopsy within 24 h postmortem, from a subject with a non-cardiovascular cause of death. This specimen was rinsed in DPBS and stored in the same manner. Tissue collection was performed according to protocols sanctioned by the Ethics Board at Erasmus MC (MEC 2008-147 for carotid endarterectomy samples, MEC 2017-300 for the autopsy sample).

### Sample Preparation

**Figure 1:**
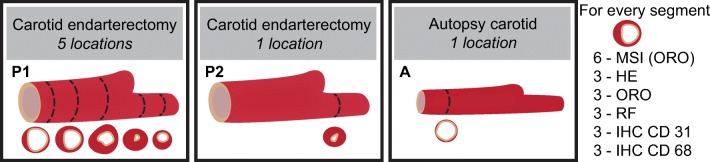
Overview of tissue samples. Five segments from carotid endarterectomy specimen 1 (**P1**), one segment from carotid endarterectomy specimen 2 (**P2**), and one segment from the autopsy carotid (**A**). Number of MALDI-MSI measurements and adjacent histological stains

One CEA specimen, referred to as P1, was transversally divided into 1-mm segments to study intraplaque variability. We hypothesize that the plaque’s molecular lipid composition may change along the length of the vessel. Five 1-mm segments, originating ≈ 3 mm apart, evenly spaced along the axial length of the CEA sample, were used for imaging. Three of the segments were located caudal to the carotid bifurcation and two segments originated from the internal carotid artery, see Figure 1. The second CEA specimen, referred to as P2, and the autopsy specimen, referred to as A, were imaged in a single axial location. The imaged 1 mm segment of P2 was located cranial to the bifurcation, in the internal carotid artery, where atherosclerotic disease stage is generally maximal. The imaged 1 mm segment of A originated from the common carotid artery, where no or minimal plaque formation is expected.

All 1 mm segments were embedded in 10% porcine type A gelatin and cryosectioned (CM3050 S, Leica Biosystems (cutting temps: OT − 21 °C; CT − 19 °C)), into 10 μm sections which were then thaw mounted onto non-conductive glass slides. After sectioning, the slides were stored at − 80 °C until imaging or histological staining. Per segment, six sections were imaged; the distance between MALDI-MSI sections was 30–50 μm. All sections were imaged or stained within 2 months after sectioning.

Prior to imaging, the slides were desiccated under vacuum at room temperature for 10 min. The matrix, 2,5-dihydroxybenzoic acid (DHB), was applied using sublimation (home-built sublimation system as described in Dekker et al. [22]) at 125 °C for 10 min; 50 mg of DHB was dissolved in 5 ml acetone. No washing, re-crystallization, or addition of ionization agents was used.

### Histological Staining

Histological staining was performed on adjacent slides, which were histochemically stained by Hematoxylin-Eosin (HE), Oil Red O (ORO), and Resorcin-Fuchsin (RF) staining, to visualize general structure, lipids, and collagen and elastin, respectively. Immunohistochemical (IHC) staining was performed to visualize endothelial cells and macrophages. The slides imaged with MALDI-MSI were stained by ORO after MSI.

We assessed the plaque tissue according to the plaque classification scheme of Virmani et al. [2, 3] and examined the tissue for features of plaque vulnerability (i.e., necrotic core, calcification, thrombus). We also assessed the tissue’s structural variability over consecutive tissue sections that originate from the same 1 mm segment. We used the histopathological data as a guidance to interpret the MALDI-MSI data.

### Data Acquisition

MALDI-MSI was performed on a MALDI-q-TOF mass spectrometer (Waters Synapt G2Si-TOF, Manchester, UK), operated in positive mode, at the instrument’s “resolution mode” (single-pass reflectron TOF) with a spatial resolution of 50 μm. An Nd:YAG (355 nm) laser was operated at 2000 Hz, at 50% of the maximum laser energy available (250/500 laser energy). At this energy level, the laser spot was ≈ 40 μm in size. The mass range was 300–1200 *m/z* and the scan time per pixel was 0.5 s using a left-right scanning pattern. The data was acquired using MassLynx v4.1 software (Waters, Manchester, UK), and HDimaging v1.4 software was used to export the data to imzML format. Prior to imaging, the machine was calibrated with red phosphorus spotted onto a steel plate.

### Mass Identification

For the identification of the various lipid components, lipids were extracted [23] by homogenizing 5 mg of tissue in 250 μL of Milli-Q water. The tissue was taken between sections P1–4 and P1–5. Ten microliters homogenized sample was combined with 10 μL 10% TEA solution (trimethylamine (10/90, *v*/*v*) in methanol/dichloromethane (DCM) (50/50, *v*/*v*)) and mixed thoroughly. Subsequently, 450 μL methanol/DCM (50/50, *v*/*v*) was added to this mixture. Samples were incubated on the roller bank for 30 min at 4 °C. After incubation, samples were centrifuged at 14,000 rpm for 20 min at 4 °C. Supernatant was transferred to a glass vial, and freeze dried until further use.

The extract was subjected to high-resolution MS for accurate mass data and MS/MS experiments for structural information, as described in Angel et al. [24]. Ten microliters of the extract was mixed with 190 μL of a methanol/chloroform (1/1, *v*/*v*) mixture and infused at a rate of 240 μL/h. In a second set of experiments, ammonium acetate was added to efficiently detect the cholesteryl esters (CEs), 10 μL of the extract was mixed with 180 μL of a methanol/chloroform (1/1, *v*/*v*) and 10 μL of a 10 mM ammonium acetate solution was added; the cholesteryl esters appear as NH_4_^+^ adducts [25].

High-resolution mass data were obtained with an LTQ Orbitrap XL mass spectrometer with an electrospray ion source operated in the positive ion mode using the following settings: source voltage 4~kV and capillary temperature 300 °C. In such experiments, the analyte molecules appear as [M + H]^+^ or as [M + NH_4_]^+^ species, whereas in MALDI imaging experiments, they are usually cationized by residual Na^+^ and/or K^+^. Peaks within 2 ppm of the expected *m/z* values were taken into consideration for identification by MS/MS experiments.

For lipids such as the phosphatidylcholines (PC) and sphingomyelins (SM), product ions in MS/MS spectra (for example *m/z* 184.1) may lie below the cut-off mass of the ion trap (27% of precursor mass). To circumvent such discrimination effects and to reliably identify such species, MS/MS spectra were recorded on a triple quadrupole mass spectrometer, in our case an AB Sciex API-3000 mass spectrometer operated in the positive ion mode, using the following settings: source temperature 200 °C, capillary voltage 5.5 kV, and collision energy 20 V.

Many of the ionized lipids undergo a common dissociation reaction giving a common product ion, for example *m/z* 369.3 for the CE and *m/z* 184.1 for PC and SM. In such cases, a precursor ion scan (i.e., a scan of MS1 at fixed MS2) allows the identification of the CE, PC, and SM precursor ions. Thus, both PC and SM components are identified by setting MS2 at *m/z* 184.1, and then scanning MS1, whereas the CE molecules are identified by selecting MS2 at *m/z* 369.1, followed by an MS1 scan. Such experiments were performed to identify all CE components using cholesteryl heptadecanoate (CE 17:0) as internal standard as described by Liebisch et al. [25]. The identified peaks were matched with the internally calibrated MSI data (Figure 3) and identification was deemed positive if the masses agreed to within 2 ppm. Identified PCs, SMs, and CEs are listed in supplementary information A.

### Development of Data Processing Pipeline

MSI data in the form of imzML files were loaded into MATLAB™ 2017a (Natwick, USA) unbinned, resulting in dataset with 20,000 to 40,000 pixels per section, depending on the imaged area, and 79,413 mass bins. Data processing steps that were used to reduce this complex dataset and retain the lipid spectrum exclusively are described below.

### Pre-Processing

#### Spectral Processing Imaging Data

**Figure 2:**
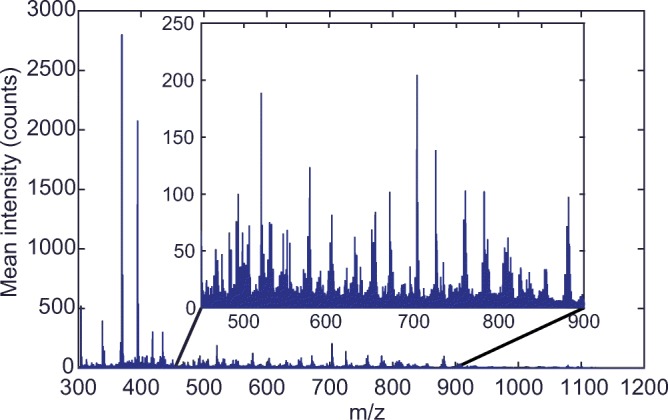
Typical MALDI-MSI spectrum P1–4 calibrated, mean spectrum, non-normalized

**Figure 3:**
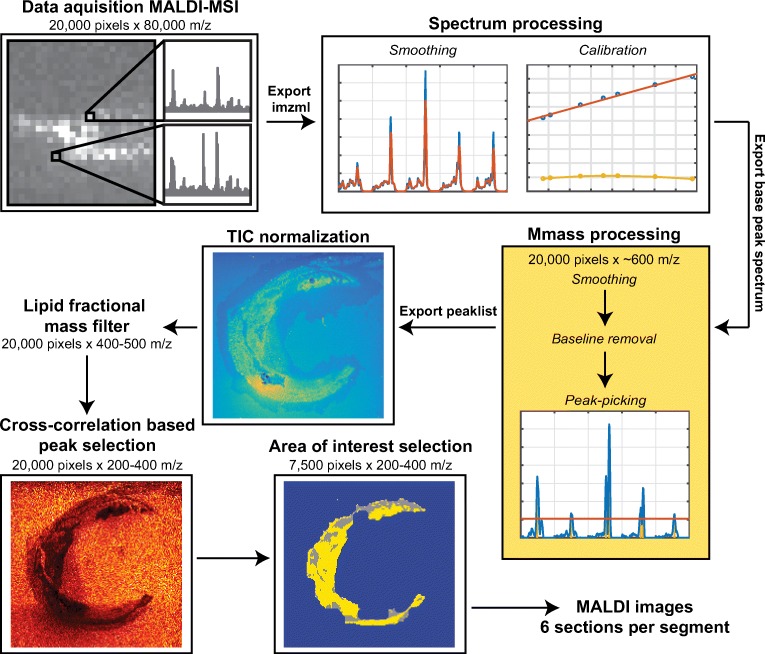
Flowchart of MSI data processing, showing the consecutive data processing steps of the MSI data. The number of m/z data points is reduced from 80,000 to 200–400 by peak picking, elimination of non-lipid peaks, and cross-correlation-based background filtering. Also, the number of informative pixels is reduced from 20,000 to 7500

For each image pixel, the spectrum was smoothed using a second-order Savitzky-Golay filter with a frame length of nine data points. After smoothing, a post-measurement calibration was performed using DHB cluster peaks as lock-mass and a linear fit, see Figure 3. In Figure 2, a representative mean spectrum of calibrated MALDI-MSI data is shown.

#### Peak-Picking

We perform peak-picking to make a pre-selection of the most important features of the data, for which we used the base peak (BP) spectrum because of the heterogeneous nature of our samples. The BP spectrum shows for each *m/z* value its maximum intensity measured over the entire MSI dataset [26]. Thus, the BP spectrum allows for detection of high-intensity lipids that are only present in a small region. After calibration, the BP spectrum was computed for the entire image and exported to mMass v5.5.0 [27]. We performed peak-picking in mMass by: (1) smoothing (Savitzky-Golay filter: window size 0.1 *m/z* and 2 cycles), (2) baseline correction (precision 15, relative offset 25), and (3) peak-picking (S/N threshold 10.0, peak-picking height 80%), see Figure 3. The filter settings used in this process were chosen heuristically: the additional peaks that are selected with less restrictive parameter settings are found to be removed in later processing steps. This supports the internal consistency of the workflow we propose here. The peak list was exported as a text file.

### Imaging Data Processing

#### TIC-Normalization and Peak Matching

The calibrated imzML measurement and the peak list were imported into MATLAB™ (The MathWorks, Natick, MA, USA). The data was normalized using total-ion current (TIC) normalization; see Figure 3. The peak list exported from mMass was used to make a pre-selection and to that aim, the peaks of the mMass-derived peak list were matched to the MSI data. The peak list was generated in mMass because the mMass peak-picking algorithm is better suited to process mass spectrometry data than the MATLAB findpeaks algorithm. For instance, in mMass, the noise level is calculated locally and adjusted for every *m/z* value, which results in fewer false-positive peaks compared to the findpeaks algorithm of MATLAB.

Because the data processing pipeline was developed in MATLAB, the peaks of the mMass-derived peak list were matched to the MSI data imported in MATLAB. Using the findpeaks algorithm of MATLAB™, the peaks in the mean TIC normalized spectrum of the image were detected. Subsequently, these peaks were matched to the peaks found in the mMass BP spectrum with a mass tolerance of ± 0.02 *m/z*; this resulted in ≈ 600 peaks. The data was subsequently binned around these peaks, with a bin width of ± 1 data point. The sample pitch of the TOF mass analyzer used varied with mass-to-charge ratio, increasing from ± 0.0075 *m/z* at 300 *m/z* to ± 0.015 *m/z* at 1200 *m/z*.

#### Lipid Fractional Mass Filter

**Figure 4:**
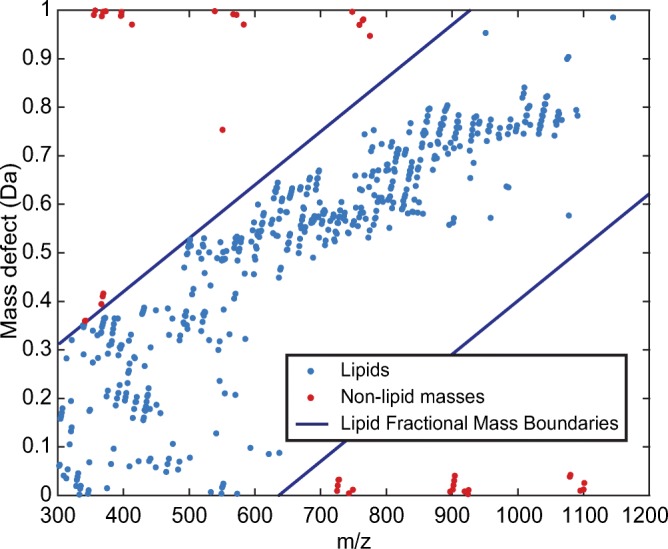
Lipid fractional mass filter. Masses outside the lipid fractional mass boundaries cannot be lipids

At this point, the dataset contains only the 600 most intense *m/z* peaks, which includes the lipids of interest as well as background and other non-lipid ions. To eliminate these unwanted signals, we applied several data reduction steps, aiming to maintain only lipid-representing *m/z* values. First, we applied a lipid fractional mass filter [26, 28] to remove non-lipid peaks from the dataset. Masses that have a fractional mass outside the lipid fractional mass boundaries cannot be lipids and are therefore discarded; see Figure 4.

#### Cross-Correlation-Based Peak Selection

Subsequently, we performed peak selection based on background peaks to remove non-biological *m/z* values, similar to Fonville et al. [29]. In this method, the correlation of images with a background reference peak was used to find matrix-related peaks and embedding medium-related peaks. Only masses that correlated negatively with this background reference mass were retained. The reference mass was chosen upon visual inspection; for all samples, we used 419.2 *m/z* as the background reference mass; see Figure 4 step 5. Features that are important for choosing a good reference mass are: (1) high intensity in background area, (2) low intensity in tissue area, and (3) smooth transitions between the two areas.

We use the background reference mass and compute the cross-correlation using the Pearson correlation coefficient:

$$ {R}^2=\frac{\sum \Big(\left({x}_i-\overline{x}\right)\left({y}_i-\overline{y}\right)}{\sigma_x{\sigma}_y} $$

We then took the 9 highest correlations to this reference mass and used these to calculate the reference to all masses in the spectrum. We then calculated the mean correlation of all these 10 references. If this was negative, a mass would be marked as being non-background and retained. This step reduced the number of *m/z* values to 200–400 peaks.

For sections with highly calcified regions, as were present in P1, an extra processing step was necessary due to noise allocated to the presence of these regions. This additional step is further explained in the supplementary information B.

#### Area of Interest Selection

**Figure 5:**
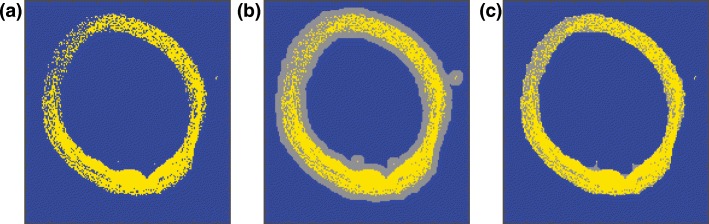
Pixel classification. (**a)** Pixel selection based on K-means clustering (yellow) (*N* = 2). (**b)** Dilated pixel selection to ensure continuity (gray). (**c)** Final continuous pixel selection

After elimination of the background and non-lipid peaks, we used a K-means clustering algorithm with *N* = 2 clusters to identify lipid-rich tissue areas; see Figure 5a. Small gaps in the data were filled by first dilating the positive structures in the image with a six-pixel circular kernel (MATLAB™ function imdilate); see Figure 5b, and subsequent removal with the same kernel (imerode), resulting in a continuous region of interest for lipid analysis; see Figure 5c.

This selection of pixels containing lipid-rich tissue was additionally used to remove the last background peaks, by discarding masses that were more intense in the background pixels than in the lipid-rich tissue pixels. The combination of data processing steps reduced the datasets to 3000–14,000 pixels, depending on section size, and 200–400 *m/z* values per measurement.

### Reproducibility

With the data processing pipeline presented here, we intend to compare lipid profiles along the length of the carotid artery within a patient and between different patients. Therefore, we need to estimate the variability between experiments and the biological variation within the samples.

The coefficient of variation (CV) is a standardized measure for the variability in relation to the mean of the population and was previously applied in MSI by Tillner et al. [30, 31]. It is defined as the ratio of the standard deviation of a population (σ) to the mean of a population (μ): $\mathrm{CV}=\frac{\upsigma}{\upmu}\times 100$, expressed as a percentage. CV is invariant to the size of the population which makes it applicable to our relatively small dataset of six sections per segment [32]. Also, CV is applicable to data with a wide variation in mean values, which is the case for our MSI dataset. The CV represents population variability: the lower the CV in a set of repeated experiments, the higher is the reproducibility of the MSI measurement.

The quantitative reproducibility of these MSI datasets depends on the repeatability of the measurement, which includes instrument parameters but also the stochastic ionization process and efficiency of transfer into the analyzer, the heterogeneity in the sample as prepared for imaging, and variability between the sections within one segment. We assessed the baseline reproducibility of the instrument by spotting two lipid standards on top of homogenized carotid artery tissue. Two microliters of ≈ 1000 pmol/μL PC 17:0/17:0 and 2 μL of ≈ 1560 pmol/μL CE 17:0 were spotted onto the homogenate. Both lipid standards were spotted and measured six times with the instrument settings reported in the “Materials and Experimental Methods” section and from that data, we calculated the CV value.

After data reduction, the tissue image dataset contains 200–400 *m/z* values; however, this data selection still contains isotopes. When calculating the CV, we used the de-isotoped *m/z* values (≈ 160) to avoid bias of similar isotope peaks. Every segment contains six tissue sections of which we calculate the mean intensity of the lipid-rich pixels for every *m/z* value. Resulting in 6 × 160 mean intensities, making up 160 populations for which we calculate the CV. We then calculated the mean CV value for every dataset ($\overline{CV}$) to allow comparison between the different segments.

We used the histopathology images to verify if all six sections within one segment were the same from a morphological perspective.

## Results

### Reproducibility

We found that the CV of the measurement of the lipid standard PC (17:0/17:0) is 10% for [M + H]^+^, 8% for [M + Na]^+^, and 16% for [M + K]^+^. For cholesteryl esters, we only looked at [M + Na]+, since it is the only ion that is stable in time of flight [33], and we found a CV of 16%. The relative intensity of the PC [M + H]^+^ peak is about 100 times higher than that of PC [M + K]^+^ and CE [M + Na]^+^, which have about the same relative intensity. This means that PC [M + H]^+^ is less susceptible to the influence of noise on the signal, explaining the lower CV value.

In the imaging data, tissue structure and content was not apparently different in the series of histologically stained sections within 1 mm segments. We thus assume that the CV values per segment represent MALDI-MSI measurement variability and not biological variability.

**Figure 6:**
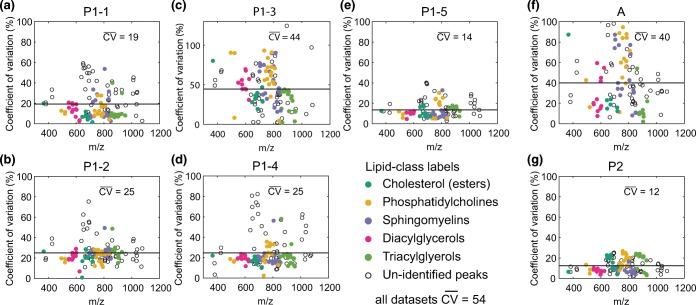
Coefficient of variation calculated using the mean intensity of every *m/z* value and comparing these values over all six measurements per segment. The mean CV value across the *m/z* values is given and depicted as a horizontal line. The different assigned lipid classes have been clustered and are color coded. **(a)** P1–1. **(b)** P1–2. **(c)** P1–3. **(d)** P1–4. **(e)** P1–5. **(f)** A. **(g)** P2

Figure 6 shows the CV distribution of all measured segments. P2 shows the lowest $\overline{CV}$of 12%, also P1–1, P1–2, P1–4, and P1–5 show relatively limited variability ranging from 14 to 25% for $\overline{CV}.$ Furthermore, P1–1, P1–2, and P1–4 show some *m/z* values with a higher CV; most of these peaks could not be identified. P1–3 and A show significantly higher $\overline{CV}$values of 44% and 40%, respectively. Computing the total variability across all peaks in all datasets without matching the segments, results in a $\overline{CV}$ of 54%.

Furthermore, we expected to find that $\overline{CV}$ would be lower when only comparing adjacent sections (30–50 μm apart), since biological variation between these sections is relatively minimal. Also, we hypothesized that two sections that were measured on the same glass slide, and thus have had the exact same sample preparation and storing conditions, would show a lower $\overline{CV}$. However, we did not find these conditions to influence $\overline{CV}$.

### Relation Between Lipid Content and Plaque Phenotype—Preliminary Observations

We hypothesize that the lipid composition of a plaque reflects plaque phenotype, and therefore expect to find specific lipid signatures in phenotypically different plaque structures. In our dataset, we could examine thrombus, a key component in ischemic events, and changes in lipid profile occurring with development stage.

#### Thrombus

**Figure 7:**
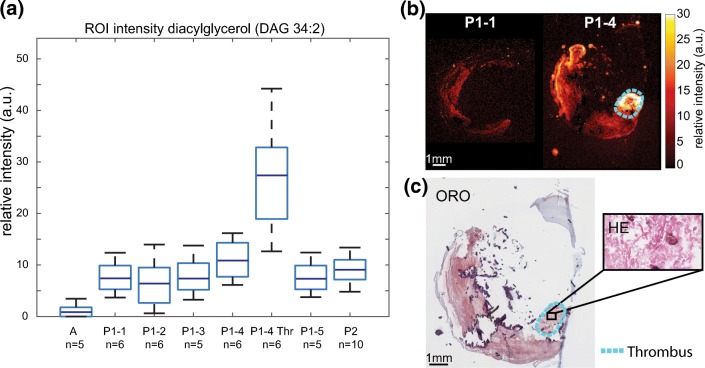
Distribution of diacylglycerol (DAG) 34:2 (*m/z* 575.51). (**a)** Boxplot of average intensity of diacylglycerol (DAG 34:2, [M + H-H_2_O]^+^) in a 300 × 300-μm region of interest in tissue segments. Expression in the thrombus-containing ROI of segment P1–4 (P1–4 Thr) is highly elevated. (**b)** MALDI MSI of P1 segment 1 and 4. The thrombus region is outlined in cyan. (**c)** ORO staining of MALDI section and HE staining of adjacent section indicating thrombus

Segment P1–4 contained thrombus; see Figure 7c, in which monoacylglycerols (MAG), diacylglycerols (DAG), and triacylglycerols (TAG) were abundantly present. When we compared 300 × 300 μm regions of interest (ROIs) in tissue segments, we found the DAGs to be more abundant in the thrombus region of P1–4 compared to another region in the same segment and also compared to other segments and other tissue samples. As such, a high concentration of DAGs appears to be thrombus-specific. In Figure 7, an example of the distribution and intensity of DAG 34:2 is shown, in comparison to the other tissue sections measured. In the supplementary information C, a list of all peaks assigned as DAGs with distributions similar to DAG 34:2 are given.

#### Plaque Development Stage

**Figure 8:**
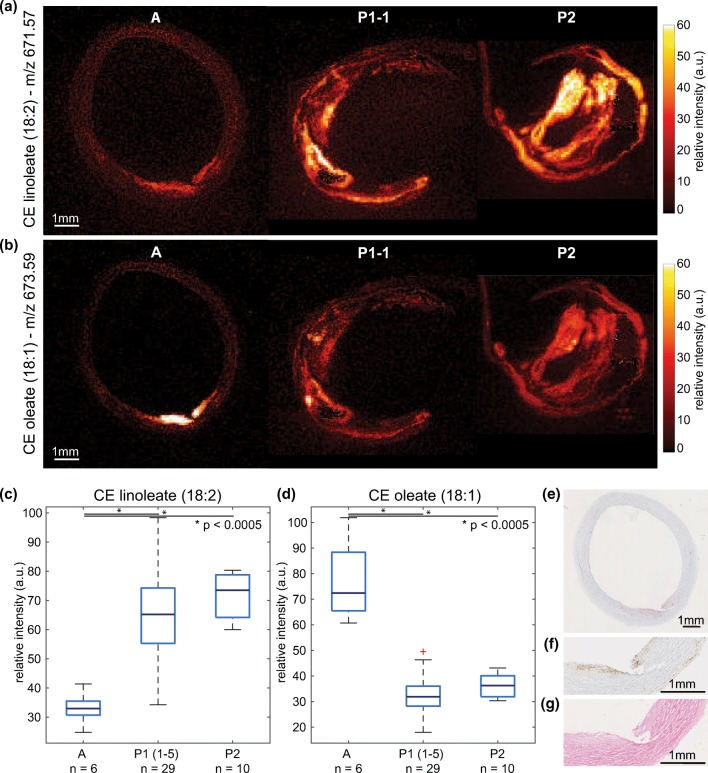
Distribution of cholesteryl linoleate (*m/z* 671.57) and cholesteryl oleate (*m/z* 673.59). (**a)** MALDI MSI of CE 18:2 in autopsy sample, P1–1, and P2. (**b)** MALDI MSI of CE 18:1 in autopsy sample, P1–1, and P2. **c** Box plot of maximum intensity (99th percentile) cholesteryl linoleate (CE 18:2), with over-expression in advanced plaques (P1 and P2), *p value < 0.0005. (**d)** Box plot of maximum intensity (99th percentile) cholesteryl oleate (CE 18:1), with over-expression in early plaque (autopsy), *p value < 0.0005. (**e)** Oil Red O staining of MALDI MSI section, showing fatty streak. (**f)** Immunohistochemistry CD68 stain (zoom, adjacent section), showing macrophages in fatty streak. (**g)** HE stain (zoom, adjacent section), showing fatty streak

The autopsy sample (sample A) contained a small fatty streak, which is an early manifestation of atherogenic changes in the vessel wall, as can be seen on the histological staining; Figure 8e–g. This sample was marked as an early-stage plaque whereas the two CEA samples, P1 and P2, were advanced atherosclerotic plaques. We compared the differences in abundance of prominent lipid peaks between early- and late-stage disease and found that the early lesion is significantly enhanced in cholesteryl oleate, whereas the advanced plaques exhibit higher concentrations of cholesteryl linoleate, see Figure 8a and c.

## Discussion

In this study, we composed a framework to investigate the lipid content of human carotid atherosclerotic plaques. Arterial tissue was systematically processed for both MSI and histology, ensuring that MSI findings could be contextualized in terms of histopathology. Also, the data-processing pipeline presented here can effectively and reproducibly assess the complex MALDI-MSI dataset in a way that focuses on the lipid distribution in the relevant tissue locations.

Our protocol differs from previous MALDI-MSI studies on human atherosclerotic tissue [12, 16–18] in the following ways. To our knowledge, this is the first study in which multiple carotid artery plaque specimens have been measured by MALDI-MSI. Based on this collection of carotid artery measurements, we optimized our data processing protocol. We have observed that the settings for discriminating tissue signal from background signal by negative cross-correlation are dependent on the composition of the tissue investigated, in particular the presence of calcifications. We expect that this data-processing step may need to be adjusted when investigating other arterial beds, and the method allows these adjustments. Secondly, this is the first MSI study on arterial tissue that allows measuring both differences between- and within-specimen lipid content and determines intra and inter variations in the tissue. Finally, we quantified the reproducibility of the samples.

We designed the data processing pipeline to be easily adjustable for other MALDI-MSI research purposes. We processed the data to the standard imzML format, which is supported by all MS instruments. Moreover, the processing pipeline is generated in mMass and MATLAB, which are both software packages in common use and easily available. The design of our processing pipeline makes the method applicable and adjustable for research in other labs.

We quantified the variability of the MALDI-MSI measurements by determining the coefficient of variation for the six tissue sections measured per segment. $\overline{CV}$ varied between 12 and 44%, with an average value of 25%. Two segments, P1–3 and A, showed relatively larger variability which may be attributed to different factors. In the case of segment P1–3, there were large calcifications present in the sample. This influenced the quality and amount of tissue per glass slide, which can be seen from the K-means pixel segmentation of these sections shown in the supplementary information D. These large variations in sections most likely contribute to an elevated CV.

In the case of segment A, this tissue is biologically different from P1 and P2, because it also contains the outer vessel wall layers, i.e., the tunica media and tunica adventitia. These layers are typically not as lipid-rich as the intimal layer. Therefore, the MALDI-MSI signal is much lower in these areas, resulting in a lower mean intensity value. When the mean values in the population are smaller, the CV becomes more sensitive to relatively small changes. Also, differences in sample collection may have had an—unknown—impact. These results indicate that, in sections of lipid-rich atherosclerotic plaque, this protocol allows us to achieve a quantitative accuracy of approximately 12–20% for abundant lipids, which sets a lower limit for the detection of individual, local, or longitudinal variations in lipid profile.

Our analysis of three carotid plaque samples indicates that abundance of specific subtypes of cholesteryl esters may be related to atherosclerotic disease stage. Several studies have associated the presence of cholesteryl oleate (*m/z* 673.59) with early-stage plaques [34–39]. Our MALDI-MSI data also showed significantly higher cholesteryl oleate intensity in the early plaque of sample A, compared to the advanced plaques in the CEA tissue specimens. In addition, cholesteryl linoleate (*m/z* 671.57) has been shown to be more abundant in advanced plaques, compared to early-stage plaques [40]. While our limited number of samples does not allow us to draw quantitative conclusions, strikingly, we find cholesteryl oleate-to-linoleate ratios that are in close agreement with previous literature [41].

In our dataset, we also observed relatively high signal of DAG species in thrombus tissue. With the current dataset, we cannot rule out the possibility that these DAG molecules represent fragmented TAGs. However, relative intensities of MALDI-MSI data of DAG molecules were different for thrombus compared to other segments, whereas their presumed TAG precursor molecules did not show this trend, indicating that we might be observing original DAG molecules, not TAG fragments. DAGs have at least two roles in biology, first, as an intermediate molecule for the synthesis of TAG and phospholipids, and second, as a regulator of protein kinase C (PKC) isozymes, which modulate several cellular processes [42]. Accumulation of DAGs has been associated with diabetes, cancer, and cardiovascular disease [43]. PKC isoforms have also been associated with platelet activation and phosphatidylserine (PS) exposure [44], underlining our finding of a high abundance of DAGs in a thrombus.

In this study, we imaged six sections per segment and quantified the reproducibility of these sections, which is indicative for the minimum fold change in intensity necessary to be significant. In future studies, in which we plan to investigate a larger collection of specimens, it is therefore possible to reduce the number of sections per site that we measure. Together with the semi-automated analysis presented here, and technological advances such as continuous scanning modes, this makes it feasible to perform systematic analyses of molecular lipid composition in larger sets of atherosclerotic specimens.

In conclusion, we have developed a protocol for MALDI-MSI measurements of lipids in human carotid plaque tissue using a new analysis pipeline. This method can now be applied to larger collections of carotid plaque specimens, allowing to assess whether the carotid plaque’s lipid signature is correlated to plaque phenotype and vulnerability. If so, this opens up new opportunities in terms of diagnosis and patient stratification and could enable the investigation of the effect of lipid-lowering medication on plaque composition and rupture-risk.

## Electronic Supplementary Material

ESM 1 (DOCX 2787 kb)
