# Supplementary material for: Data Processing Pipeline for Lipid Profiling of Carotid Atherosclerotic Plaque with Mass Spectrometry Imaging
Source: J Am Soc Mass Spectrom. 2019 Jun 27;30(9):1790–800. doi: 10.1007/s13361-019-02254-y (PMC6695360; doi:10.1007/s13361-019-02254-y)
Supplement: Supplementary file 1 — (DOCX 2787 kb) [file 13361_2019_2254_MOESM1_ESM.docx]

Supplementary information: Data processing pipeline for lipid profiling of carotid atherosclerotic plaque with mass spectrometry imaging

Mirjam Visscher^1,^*,Astrid M. Moerman^1,^*, Peter C. Burgers^2^, Heleen M.M. Van Beusekom^3^, Theo M. Luider^2^, Hence J. M. Verhagen^4^, Antonius F. W. Van der Steen^1,5,6^, Kim Van der Heiden^1,^* and Gijs Van Soest ^1,^*

* These authors contributed equally

1. Department of Biomedical Engineering, Thorax Center, Erasmus MC, Rotterdam, The Netherlands
2. Department of Neurology, Laboratory of Neuro-Oncology, Erasmus MC, Rotterdam, The Netherlands
3. Department of Experimental Cardiology, Thorax Center, Erasmus MC, Rotterdam, The Netherlands
4. Department of Vascular and Endovascular Surgery, Erasmus Medical Center, Rotterdam, The Netherlands
5. Medical Delta, Delft, The Netherlands
6. Shenzhen Institutes of Advanced Technology, Chinese Academy of Sciences, Shenzhen, China

# A Supplementary information: Identified lipids in atherosclerotic plaque

Table S 1. Identification of phosphatidylcholines (PC) and sphingomyelines (SM) by high resolution mass spectrometry (orbitrap) and MS/MS (triple quadrupole)

| Experimental mass^a^ | Calculated mass [M+H]+^b^ | Delta (ppm)^c^ | Identification by MS^2 d^ |
| --- | --- | --- | --- |
| 369.35175 | 369.35156 [M-H_2_O+H]+ | -0.5 | cholesterol |
| 703.57471 | 703.57487 | 0.2 | SM 16 18 1 |
| 725.55659 | 725.55922 | 3.6 | Identified as [M+Na]+ |
| 731.60597 | 731.60617 | 0.3 | SM 18 18 1 |
| 734.56912 | 734.56938 | 0.4 | PC 16 16 0 |
| 758.56916 | 758.5694 | 0.3 | PC 16 18 2 |
| 760.58483 | 760.58504 | 0.3 | PC 16 18 1 |
| 780.55191 | 780.55373 | 2.3 | Identified as [M+Na]+ |
| 782.56889 | 782.56938 | 0.6 | PC 18 18 4 |
| 784.58494 | 784.58503 | 0.1 | PC 18 18 3 |
| 786.60048 | 786.60068 | 0.3 | PC 18 18 2 |
| 788.61586 | 788.61633 | 0.6 | PC 18 18 1 |
| 810.60037 | 810.60068 | 0.4 | PC 18 20 4 |
| 813.68395 | 813.68437 | 0.5 | SM 18 24 2 |

1. Experimental mass (Orbitrap) for protonated species
2. Calculated mass (http://www.lipidmaps.org/tools/structuredrawing/GP masscalc.php) for protonated species
3. Difference calculated and experimental mass (in ppm)
4. Identification by exact mass and MS/MS data (triple quadrupole). Numbers refer to chain lengths and number of unsaturations (e.g. SM 16 18 1 represents a sphingomyeline with one C16 and one C18 chain and one double bond; position double bond(s) not determined)

Table S 2. Identification of phosphatidylcholines (PC) and sphingomyelines (SM) Na^+^ adducts by high resolution mass spectrometry (orbitrap) and MS/MS (triple quadrupole)

| Experimental mass^a^ | Calculated mass [M+Na]+^b^ | Delta (ppm)^c^ | Identification by MS^2 d^ |
| --- | --- | --- | --- |
| 725.55659 | 725.5568 | 0.3 | SM 16 18 1 |
| 780.55191 | 780.5514 | -0.7 | PC 16 18 2 |

1. Experimental mass (Orbitrap) for Na^+^ adduct
2. Calculated mass (http://www.lipidmaps.org/tools/structuredrawing/GP masscalc.php) for Na+ adduct
3. Difference calculated and experimental mass (in ppm)
4. Identification by exact mass and MS/MS data (triple quadrupole). Numbers refer to chain lengths and number of unsaturations (e.g. SM 16 18 1 represents a sphingomyeline with one C16 and one C18 chain and one double bond; position double bond(s) not determined)


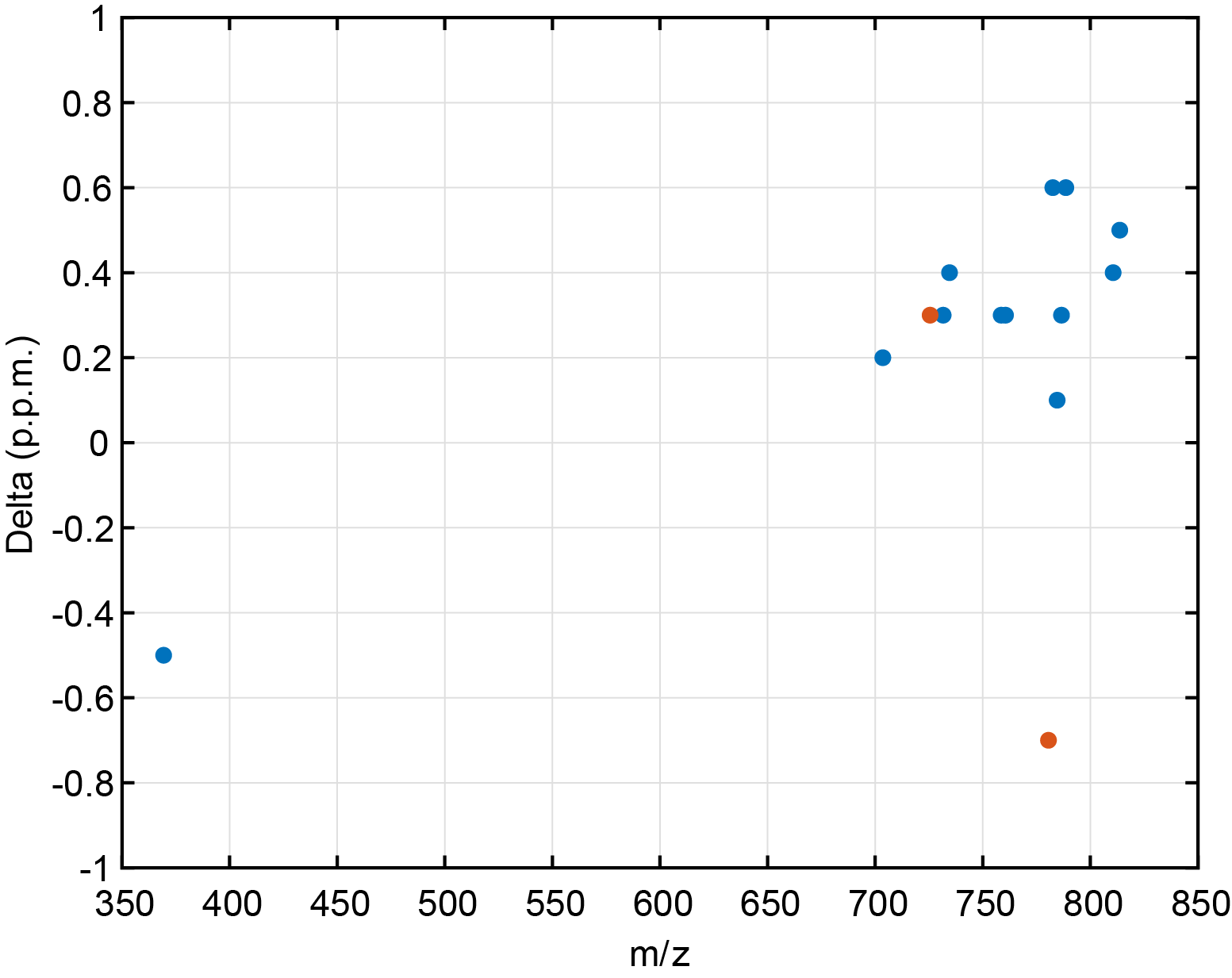


Figure S 1. Delta (ppm) as function of mass for Table S 1 and Table S 2

Table S 3.Identification of cholesteryl esters (CE) by triple quadrupole (precursor ion scan)

| Experimental mass^a^ | Calculated mass [M+NH_4_]+^b^ | Identification by MS^2 c^ |
| --- | --- | --- |
| 614.6 | 614.5870 | 14:0 |
| 628.6 | 628.6026 | 15:0 |
| 640.6 | 640.6027 | 16:1 |
| 642.6 | 642.6183 | 16:0 |
| 654.6 | 654.6183 | 17:1 |
| 656.6 | 656.6340 | 17:0 |
| 666.6 | 666.6183 | 18:2 |
| 668.7 | 668.6340 | 18:1 |
| 682.7 | 682.6497 | 19:1 |
| 684.7 | 684.6653 | 19:0 |
| 696.7 | 696.6653 | 20:1 |
| 698.7 | 698.6809 | 20:0 |
| 716.5 | 716.6340 | 22:5 |

1. Experimental mass (triple quadrupole)
2. Calculated mass (http://www.lipidmaps.org/tools/structuredrawing/GP masscalc.php) for NH_4_+ adduct
3. Peak present at mz 369.3 in precursor ion scan

# B Supplementary information: Calcifications

Inspection of the images after filtering the background peaks, the retained lipid-rich peaks revealed areas of prominent chemical noise(1) associated with calcifications in the tissue as seen in histopathology, see Figure S 2. Calcified tissue is often present in advanced atherosclerotic plaques. This hard material causes sectioning artefacts and has a limited adhesion to the glass slide. We hypothesize that the non-planarity and poor thermal coupling affect the observed peak structure.

After the first selection round, with the first reference mass, the masses remaining in the dataset are slightly different per dataset. This is due to the a-specific distributions in the images associated with calcifications, which cause the correlation of these images with our first reference mass to be located around the threshold value, causing erratic performance of this filter step. For this reason, it was not possible to select one specific reference mass to remove these peaks in all measurements. Consequently, for each measurement we determine a second reference mass by finding the most negatively correlated mass in comparison to a number of highly abundant lipids in the samples. These highly abundant lipids are stated in Table 1.

Table 1: Highly abundant lipids in all samples

| m/z | Ion | Peak assignment |
| --- | --- | --- |
| 369.35 | [M-H_2_O+H]^+^ | Cholesterol |
| 603.54 | [M-H_2_O+H]^+^ | Diacylglycerol (DAG) 36:2 |
| 671.57 | [M+Na]^+^ | Cholesteryl ester (CE) 18:2 |
| 703.57 | [M+H]^+^ | Sphingomyelin (SM) 34:1 |
| 725.56 | [M+Na]^+^ | Sphingomyelin (SM) 34:1 |
| 782.57 | [M+Na]^+^ | Phosphatidylcholines (PC) 34:1 |
| 804.55 | [M+Na]^+^ | Phosphatidylcholines (PC) 36:4 |
| 881.76 | [M+Na]^+^ | Triacylglycerol (TAG) 52:2 |

The second background mass was only accepted if it was highly anticorrelated to at least six of these eight lipids. With this second background mass, the same algorithm as with the first background mass was applied, and again only masses with a negative correlation to this second background peak were retained.


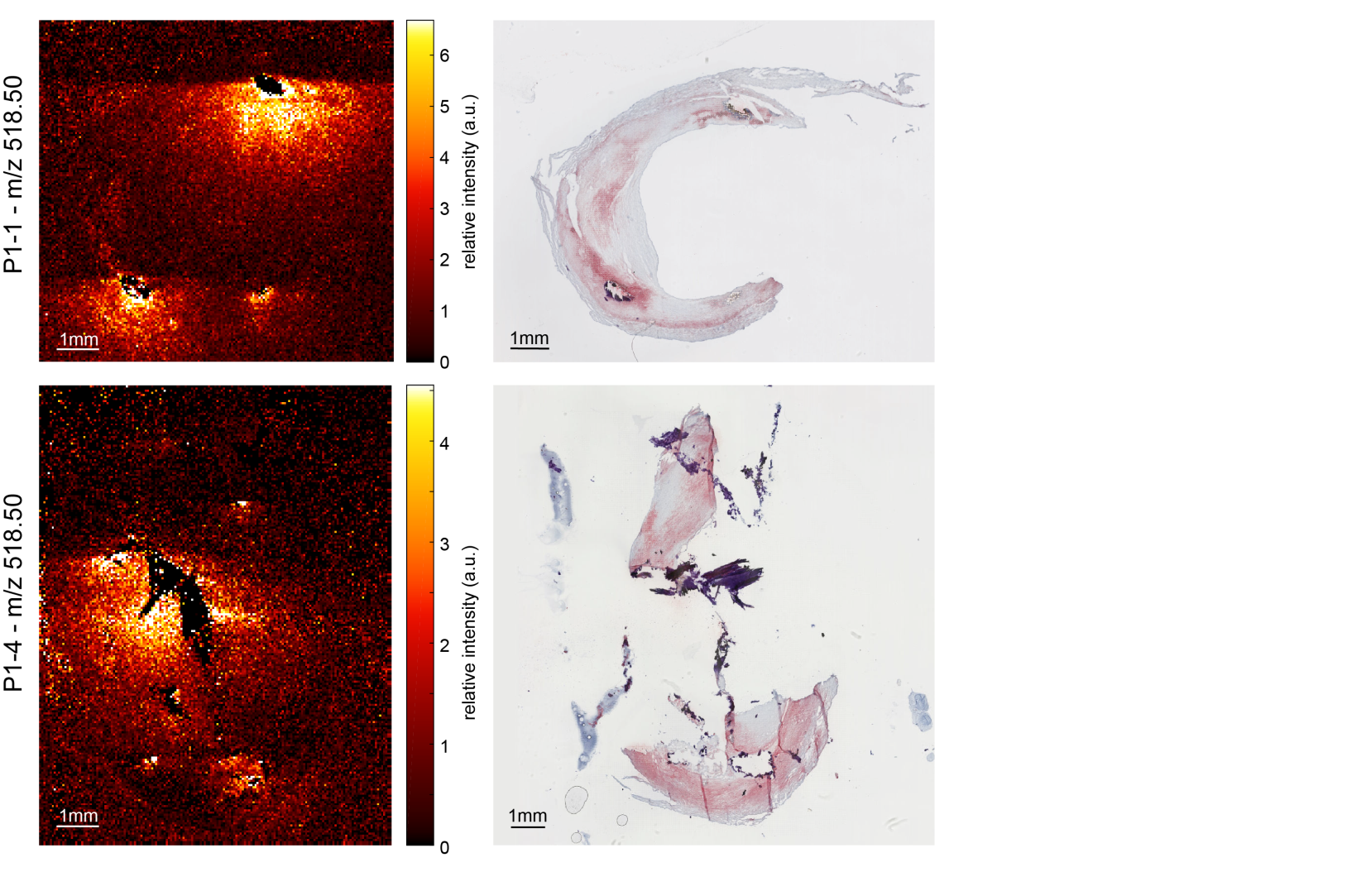


Figure S 2. Examples of noise resulting from calcified regions in P1-1 and P1-4

# C Supplementary information: Diacylglycerols in thrombus

Table S 4. Diacylglycerols (DAG) associated with thrombus area

| m/z [M-H_2_0+H]+ | Peak assignment |
| --- | --- |
| 549.49 | DAG 32:1 |
| 551.50 | DAG 32:0 |
| 573.49 | DAG 34:3 |
| 575.50 | DAG 34:2 |
| 577.52 | DAG 34:1 |
| 579.53 | DAG 34:0 |
| 599.50 | DAG 36:4 |
| 601.52 | DAG 36:3 |
| 603.53 | DAG 36:2 |
| 605.55 | DAG 36:1 |
| 607.57 | DAG 36:0 |
| 623.50 | DAG 38:6 |
| 625.52 | DAG 38:5 |
| 627.53 | DAG 38:4 |
| 629.55 | DAG 38:3 |

#
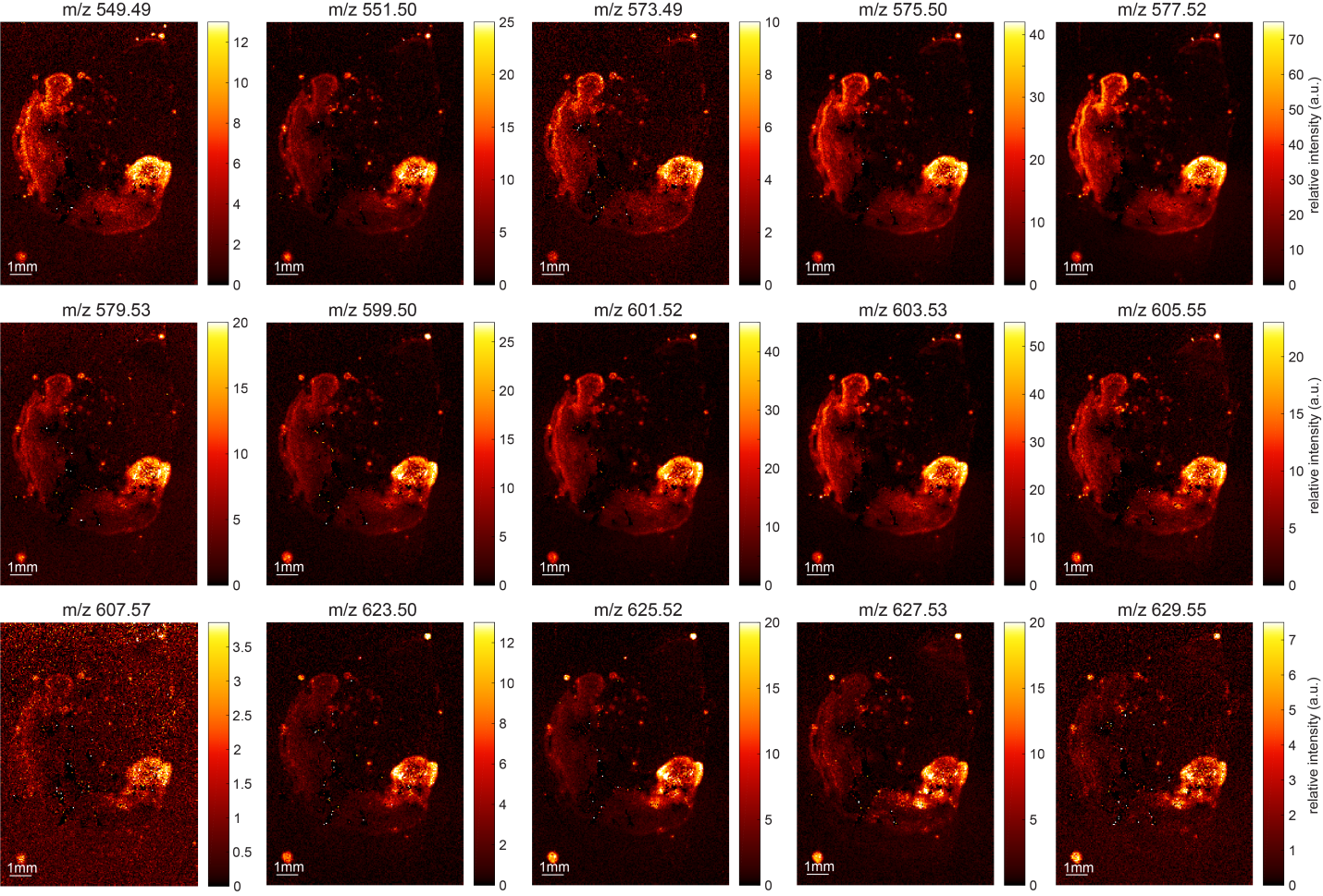


Figure S 4. Distribution of all diacylglycerols(DAG)

# D Supplementary information: Variability sections P1-3


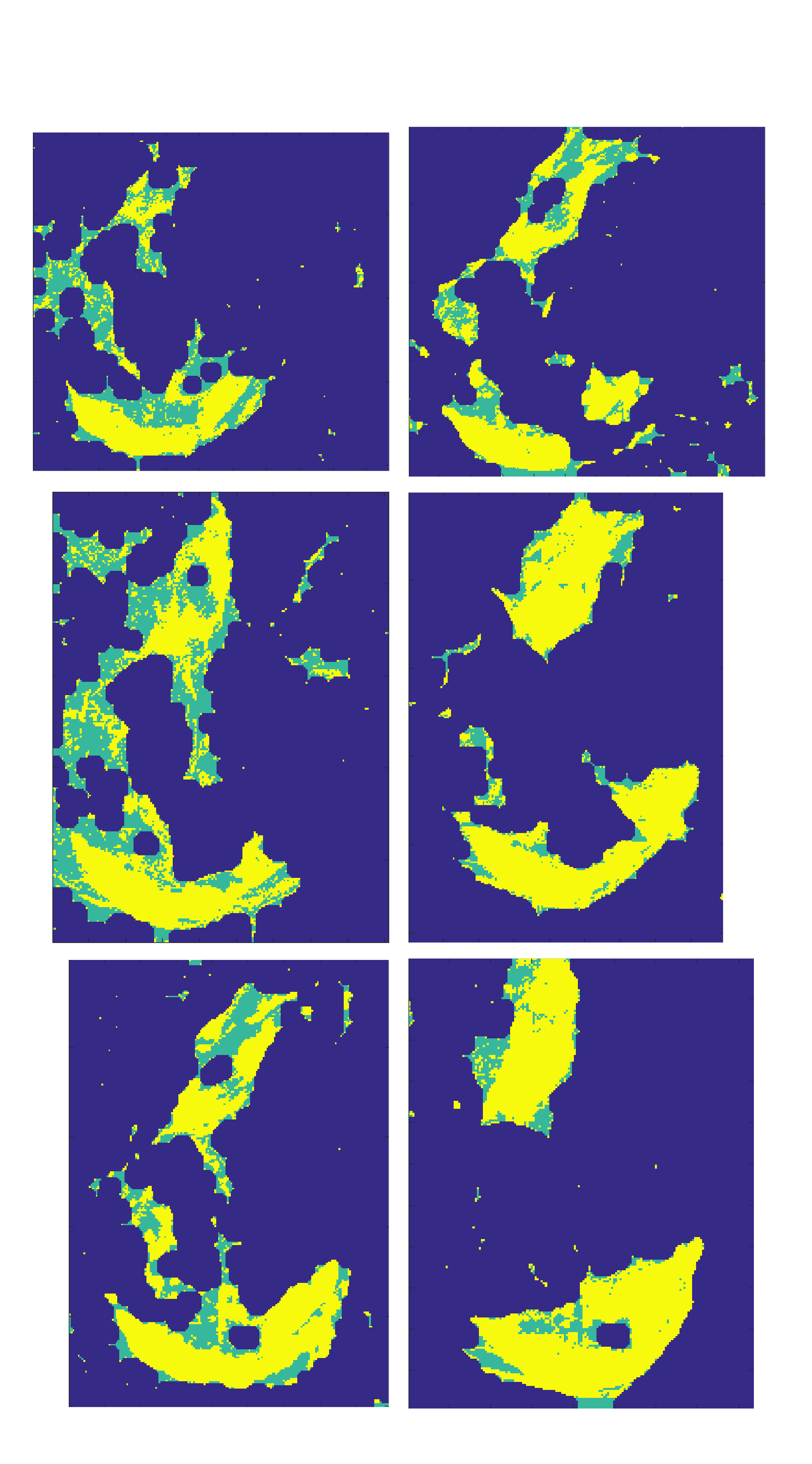


Figure S 3. Lipid-rich tissue pixels, selected by K-means, for all P1-3 sections.

1. Krutchinsky AN, Chait BT. On the nature of the chemical noise in MALDI mass spectra. Journal of the American Society for Mass Spectrometry. 2002;13(2):129-34.
